# Effect of group size and reproductive status on faecal glucocorticoid concentration and vigilance in a free-ranging population of Przewalski’s gazelle

**DOI:** 10.1093/conphys/coaa027

**Published:** 2020-04-04

**Authors:** Ruoshuang Liu, Jianbin Shi, Dingzhen Liu, Shikui Dong, Yu Zhang, Yonglin Wu, Dongsheng Guo

**Affiliations:** 1 School of Environment, Beijing Normal University, Xinjiekouwai St, Haidian District, Beijing 100875, China; 2 Key Laboratory of Biodiversity Sciences and Ecological Engineering, College of Life Sciences, Xinjiekouwai St, Haidian District, Beijing Normal University, Beijing 100875, China; 3 Administration Bureau of Mount Qilian National Park (Qinghai), Qinghai Forestry and Grassland Administration, Xichuannan Road, Chengxi District, Xining 810008, Qinghai, China; 4 Wildlife Conservation Department, Qinghai Forestry and Grassland Administration, Xichuannan Road, Chengxi District, Xining 810008, Qinghai, China

**Keywords:** *Ex situ* conservation, faecal cortisol, group-size effect, predation risk, stress level, vigilance

## Abstract

Elevated glucocorticoid (GC) concentration and increased vigilance are two common responses to predation risk in mammals. Chronic high-level GC concentration and vigilance occur at the expense of other life maintenance and reproduction activities, reflecting a trade-off between individual survival and future fecundity. Przewalski’s gazelle (*Procapra przewalskii*) is a group-living ungulate endemic to the high-altitude Qinghai Lake region of China. Group-size effect on gazelle vigilance has been examined, yet little is known about how their GC concentration is affected by group size or reproductive status. In this study, we examined the effect of group size and reproductive status on faecal glucocorticoid metabolite (FGM) concentrations and individual vigilance during different stages of the reproduction cycle (i.e. non-breeding, lambing and rutting) in free-ranging adult female Przewalski’s gazelles. Group size did not influence FGMs significantly, but mean vigilance duration increased with group size. The gazelles’ FGMs and vigilance peaked in lambing season. FGMs showed no difference between rutting season and non-reproductive season, but vigilance was lowest in the rutting season. FGMs correlated with vigilance frequency and vigilance duration. Antipredator responses of female Przewalski’s gazelles appear to change with reproductive status but not with group size in free-ranging females. Management measures should be taken in the lambing season to minimize stress on mother gazelles.

## Introduction

Reproduction is a high-cost and high-risk phase in animal life history. The trade-off between survival and reproduction is a common problem with which wild animals confront, yet it is difficult to directly measure the animals’ physiological and behavioural responses to predation (Sapolsky and Wingfield, 2003; Boonstra, 2005). The main indicators of physiological stress in mammals are glucocorticoids (GCs), namely cortisol and corticosterone (Sapolsky and Wingfield, 2003; Wingfield, 2005). GCs are released by the adrenal gland to modulate the intensity of behavioural responses to stress (Sapolsky *et al.*, 2000; Romero *et al.*, 2009). GC metabolites in faecal samples accurately reflect the concentrations of these hormones in the plasma over the preceding hours or days (Sheriff *et al.*, 2010) and avoid false results associated with capturing the animal (Schwarzenberger, 2007; Palme, 2019). For the same animal under different conditions, or conspecifics on similar diets, faecal sampling provides a relative measure of stress hormone response at different times or in different situations that can be conducted over the long term and has been widely used to monitor stress responses in wild animals including ungulates (Schwarzenberger, 2007; Sheriff *et al.*, 2011; Scott *et al.*, 2017; Kumar and Umapathy, 2019).

Vigilance is a behavioural response for detecting predators, which can indirectly improve individual fitness by avoiding predation and protecting offspring (Treves, 2000; Caro, 2005). Many studies have detected a well-known, but not universal, relationship: when group size increases, individual vigilance decreases. This negative correlation between group size and vigilance across taxa is usually explained by the ‘many-eyes effect’ or ‘risk dilution effect’, where living in larger groups reduces an individual’s probability of being preyed upon, or the ‘scramble competition effect’ where more conspecific competition for resources in larger groups causes individuals to balance the cost of vigilance and other behaviours (Lima and Bednekoff, 1999; Beauchamp and Ruxton, 2003; Fairbanks and Dobson, 2007; Beauchamp, 2008, 2017; Rieucau and Giraldeau, 2009). However, in some species and circumstances, vigilance increases as group size becomes larger, mostly because of higher intra-population aggression and competition (Shi *et al.*, 2010a; Zheng *et al.*, 2013; Beauchamp, 2016; Pecorella *et al.*, 2019). In addition, vigilance patterns may differ with seasonal changes in reproductive status (Pépin *et al.*, 2001; Richard *et al.*, 2008). Females with offspring spend more time vigilant (Liley and Creel, 2008; Rieucau and Martin, 2008; Fattorini and Ferretti, 2019), while males decrease vigilance during the rutting season for mating (Balmford and Turyaho, 1992) or spend more time guarding females and scanning for rival males (Pecorella *et al.*, 2019). Moreover, variation in vigilance is also associated with variation in adrenal activity; consequently, those factors influencing vigilance lead to changes in GCs (Sapolsky *et al.*, 2000; Goymann, 2012; Voellmy *et al.*, 2014; Blumstein *et al.*, 2016). Positive associations between mean faecal glucocorticoid metabolite (FGM) excretion and individual vigilance rates have been found in free-ranging meerkats (*Suricata suricatta*) and wild female Apennine chamois (*Rupicapra pyrenaica ornata*), suggesting context-specific links between adrenal activity and antipredator behaviours (Voellmy *et al.*, 2014; Fattorini *et al.*, 2018).

In low predation risk environments, costly and redundant antipredator responses may decline gradually in some species (Blumstein, 2002; Blumstein and Daniel, 2005; Lahti *et al.*, 2009; Christie *et al.*, 2012; Carrete and Tella, 2015; Jolly *et al.*, 2018), while retained in other species (Orrock, 2010; Shi *et al.*, 2010a; Zheng *et al.*, 2013). Antipredator responses can be innate, as well as acquired through experience and learning (Jolly *et al.*, 2018). When endangered animals are moved into captivity, predator-free enclosures or islands for conservation, their antipredator responses should be maintained, so that reintroduction to their former habitat remains a possibility (Lahti *et al.*, 2009; Orrock, 2010; Jolly *et al.*, 2018).

Przewalski’s gazelle (*Procapra przewalskii*) is a group-living ungulate in the family Bovidae. Once widespread, its range has shrunk to small separated sub-populations around Qinghai Lake in China, such that it has endangered (EN) Red List status (Li *et al.*, 2010; IUCN, 2016). Przewalski’s gazelle lives in social groups, but adult males and females are sexually segregated for most of the year and usually only come together immediately before and during the rutting seasons (Lei *et al.*, 2001). In general, they rut from late December to late January and the lambing season is from late June to early August. Adult females breed once a year and give birth to one offspring at a time. Predation on the gazelles in the wild is mostly from wolves (*Canis lupus*), while foxes (*Vulpes vulpes*) and Tibetan foxes (*Vulpes ferrilata*) may hunt newborn lambs occasionally (Li *et al.*, 2009). There have been several studies focusing on sex-specific vigilance strategies. Particularly, females increase antipredation vigilance during the lambing season, while males increase their vigilance during the rutting season when male–male mate competition peaks (Li *et al.*, 2009, 2012; Shi *et al.*, 2010b). However, the relationship between physiological responses and vigilance behaviour are poorly known.

Here, we investigated the effects of group size and reproductive status on FGM concentrations and vigilance of adult female gazelles in a free-ranging population in Qinghai Lake National Nature Reserve in China. We studied only females to avoid the confounding effect of sex on FGMs and vigilance (Li *et al.*, 2012; Palme, 2019). We examined the following: (i) whether the group-size effect on FGMs and vigilance responses were maintained in a group of free-ranging female gazelles in a predation-free enclosure; (ii) whether and how reproductive status of the female gazelles influenced their FGMs and vigilance level; (iii) how closely correlated the vigilance behaviours of the female gazelles were to their physiological response. We predict that FGMs positively correlate with vigilance.

Our main objective is to better understand how breeding females of this endangered gazelle respond to potential predation risk and the implications for the conservation and management of free-ranging populations.

## Material and methods

### Ethics statement

There was no direct contact with animals in this study, and no animals were manipulated by observers to collect the data. All the faecal samples and behavioural data were collected in compliance with Law of the People’s Republic of China on the Protection of Wildlife and the animal welfare and ethics of Beijing Normal University.

### Study site and subjects

This study was conducted at Bird Island Protection Station (99°51′22.77′′ E, 36°59′13.35′′ N) of the Qinghai Lake National Nature Reserve, located to the west of Qinghai Lake in Qinghai Province of China. Temperature data were obtained from the China Meteorological Administration and varied seasonally as follows: −23°C to 1°C in January, −9°C to 15°C in April and 6°C to 21°C in July. A habituated Przewalski’s gazelle population, rescued from the wild since 2006, was kept in a 0.81 km^2^ enclosure. The enclosure was a 2-m high wire-netting fence enclosing natural habitat, including alpine steppe and a section of river. The enclosure was wolf-free, yet a family of foxes lived within the enclosure and could get through the fence freely. One ton of hay was supplied each month from December to March. Human disturbance was limited to daily monitoring within the enclosure by one station staff each day.

The study population comprised 72 unmarked individuals in 2016, with 24 adult males, 32 adult females and 16 lambs. No animals were manipulated or handled by observers during the study period (this requires special permission from the central government agency as the gazelles are protected under China’s Wildlife Protection Law). We did not mark individuals and thus could not identify gazelles individually from physical features from a distance. Because some individuals were relocated to another enclosure by the Protection Station in February 2017, the population size reduced to 31 individuals, comprising 16 adult males and 15 adult females. This relocation provided us with an opportunity to compare the physiological and behavioural differences between the larger group in 2016 and the smaller group in 2017.

Based on previous studies, a group was defined as a herd if all individuals were within 50 m of one another (Shi *et al.*, 2010b; Li *et al.*, 2012). All the adult females and sub-adults maintained no more than 50 m from each other and displayed the same behaviours for most time during the study period, thus could be treated as one group of females. Adult males were naturally separated from females and usually at the other side of the enclosure from females. Males usually were in one or two male groups, except in the rutting season when territorial males attempted to guard the harem of females, while satellite males remained far from the females.

### Faecal sample collection and hormone analyses

Faecal samples were collected from March to April 2016, June to July 2016, December 2016 to January 2017, March to April 2017, and June to July 2017. The sampling periods corresponded to different life stages of the gazelle: spring (March–May) is the non-reproductive season, summer (June–August) is the lambing season and winter (December–February) is the rutting season.

The relocation was conducted by station staff in February 2017, and introduced stress to the relocated animals. We collected faecal samples of relocated female gazelles to validate our methods to measure FGMs. Control faecal samples of pre-relocation females were also collected from 10:00 to 12:00 on the day of the relocation. Capture of the animals began at 13:00 and was followed by transportation. The gazelles were released into a new predator-free enclosure in the evening on the same day. Faecal samples from the post-relocation females were collected again at 8:00–10:00 the next morning, ~20 h after capture. These samples were used only for validation and not in subsequent analyses.

During the study period, places where adult females defecated were located during behavioural observation of the female group in the morning. Fresh faecal samples were collected from 10:00 to 12:00 after behavioural observation and all faecal samples were collected within 2 h after defecation. This timing of collection also diminished the effect of a daily cycle on cortisol secretion (Palme, 2019). It was difficult to identify individuals; faecal samples were anonymized but were nevertheless treated as independent samples within an observation day. Impurities were cleaned from the surface of each fresh faecal sample, then samples were stored in polypropylene ziplock bags and refrigerated in a thermal box with an ice cube (500 ml) until moved into freezer in the field station at −20°C before analysis. All the faecal samples were transported frozen with ice cubes in a thermal box to Beijing, which is ~3 h away from the field station by flight (Liu *et al.*, 2018).

In the laboratory, the samples were dried with a lyophilizer at −40°C and then pulverized. Foreign materials (hair, plant fiber, soil) were removed from the samples. A very small part of each sample (0.1 g) was weighed out to be centrifuged. One millilitre of 80% methanol was added to each sample, and samples were shaken in a vortex for 30 min. After the samples were centrifuged at 5000 rpm for 10 min, supernatant from each sample was decanted into new tubes and dried using a nitrogen blowing sample concentrator, and then re-dissolved in 1 ml phosphate buffer (pH 7.2). Concentrations of FGMs were quantified using a cortisol ELISA kit (KGE008; R&D Systems, Minneapolis, MN, USA). The sensitivity of the assay was 0.071 ng/ml and intra-assay precision was <10% (Creel *et al.*, 2009; Lacasse *et al.*, 2019).

### Behaviour data

Four main behavioural states were identified—vigilance, feeding, moving and bedding—and one more category as ‘other’ behaviours that did not occur frequently (Li *et al.*, 2009, 2012; Shi *et al.*, 2010b). Vigilance was defined as an event in which the gazelle raised its head above shoulder height, with ears upright, scanning the surroundings or gazing steadily in one direction. Feeding was defined as an event where the head was below shoulder height and the individual was grazing or moving while searching between grazing bouts. Moving was characterized by walking or running with the head held no lower than the shoulders, with no foraging behaviour. Bedding was a state of lying on the ground to rest and/or ruminate. Other behaviours included grooming, defecating, fighting, mating and nursing (Li *et al.*, 2009, 2012; Shi *et al.*, 2010b).

Behavioural observations were carried out simultaneously with faeces collection. Focal animal continuous sampling and scan sampling (Altmann, 1973) were used to collect behavioural data from 08:00 to 18:00. For the focal animal continuous sampling, one focal adult female was randomly selected from the group and behaviours and their durations were recorded for 5 min. For the scan sampling, the behavioural states of all adult females in the group were scan sampled every 5 min and the number of individuals engaged in each behavioural state was recorded.

Individuals might have been sampled more than once on the same day, but it was extremely rare to sample the same individuals during one observation bout or scan because the maximum number of focal individuals selected for observations in one bout was half of the females and the focal observations were usually conducted consecutively. Temporal pseudoreplication could not be avoided because of the semi-natural environment and unmarked individuals.

### Statistical analysis

Independent *t*-test was used to compare the difference between pre-stressed faecal samples and post-stressed samples in biological validation.

Vigilance frequency (VF), percentage time spent vigilant (PVT) and mean vigilance duration (total vigilance duration divided by the number of vigilance bouts in 5 min; MVD) during 5-min observations were calculated from focal samples, while percentage of vigilant individuals (PVIs) among all group members was calculated from scan samples.

A general linear mixed model was used to detect the effects of group size and reproductive status on FGMs. The model included group size (large = 32 individuals *vs.* small = 15 individuals), reproductive status (rutting, lambing *vs.* non-reproductive) and the interactions between these variables as fixed factors. Because samples were anonymized, samples were randomly numbered between days, respectively. Sample numbers of different days were set as a random factor for repeated measurement data. Interpretation of differences among factors was supported by the least significant difference (LSD) *post hoc* method. Due to the non-normality of behavioural data, a generalized linear mixed model with Loglinear was used to identify the effect of group size (large = 32 individuals *vs.* small = 15 individuals) and reproductive status (rutting, lambing *vs.* non-reproductive) on VF with *post hoc*. Normal distribution with identity was used for MVD. For PVT and PVI, the mixed model used a gamma distribution with log. Pearson correlation was used to test for significant correlation between FGMs and vigilance behaviour. Because we could not assign faecal samples to individual females, data for correlation analyses were pooled as weekly means and set in time order (*n* = 26), with one faecal sample mean corresponding to the vigilance mean from the preceding week. All analyses were conducted with SPSS 20. Statistical significance was set at *P* < 0.05, except the *post hoc* analyses of reproductive status, in which the adjusted significance level was 0.05/3 = 0.0167 (Miller, 2012).

## Results

### Effects of group size and reproductive status on FGM concentrations

FGM concentrations (mean ± SD) of the females being relocated increased by 409%, as measured after 20 h of capture and transport (pre-relocation: 14.99 ± 2.27 ng/g, *n* = 10; post-relocation: 61.36 ± 12.46 ng/g, *n* = 8, *P* < 0.001).

The FGM concentration (mean ± SD) from individuals in the larger group in 2016 was on average 21.03 ± 0.80 ng/g (*n* = 224) and 23.71 ± 1.36 ng/g (*n* = 99) in 2017 from the smaller group. The mean FGM concentration was 14.35 ± 0.44 ng/g (*n* = 60) in the rutting season, 26.88 ± 1.09 ng/g (*n* = 174) in the lambing season and 17.07 ± 0.74 ng/g (*n* = 89) in the non-reproductive season.

There was a significant change in FGMs with change in reproductive status (*F* = 43.43, *P* < 0.001), but not with group size (*F* = 0.12, *P* = 0.730). There were no interactions between reproductive status and group size (*F* = 1.025, *P* = 0.312) (parameter estimates were in Table S1). FGMs in the lambing season were significantly greater than those in the rutting and non-reproductive seasons (*post hoc* LSD test, *P* < 0.001; Table 3), but FGM concentrations in the rutting and non-reproductive seasons were not different (*P* = 0.108).

### Effects of group size and reproductive status on vigilance

The parameters of vigilance level of the female gazelles were measured as in Table 1.

**Table 1:** Measures of vigilance parameters of Przewalski’s gazelle (mean ± SD)

	VF (no. vigilance/5 min)	MVD (s/5 min)	PVT (%)	PVI (%)
Large group	1.02 ± 0.09 (*n* = 303)	13.10 ± 1.93 (*n* = 303)	0.09 ± 0.01 (*n* = 303)	0.08 ± 0.01 (*n* = 357)
Small group	1.05 ± 0.11 (*n* = 206)	11.86 ± 1.97 (*n* = 206)	0.09 ± 0.01 (*n* = 206)	0.11 ± 0.01 (*n* = 238)
Rutting season	0.47 ± 0.10 (*n* = 70)	1.88 ± 0.36 (*n* = 70)	0.01 ± 0.00 (*n* = 70)	0.03 ± 0.01 (*n* = 86)
Lambing season	1.31 ± 0.10 (*n* = 315)	16.09 ± 1.90 (*n* = 315)	0.12 ± 0.01 (*n* = 315)	0.12 ± 0.01 (*n* = 360)
Non-reproductive season	0.64 ± 0.10 (*n* = 123)	9.29 ± 3.04 (*n* = 123)	0.06 ± 0.02 (*n* = 123)	0.06 ± 0.01 (*n* = 149)

There was significant effect of reproductive status on all vigilance parameters, but no effect of group size on VF, PVT and PVI (*P* > 0.05), except for MVD, which increased with group size (*P* = 0.027) (Table 2; parameter estimates were in Tables S2–S5).

**Table 2:** Effects of group size and reproductive status on vigilance of Przewalski’s gazelle

		*F*	*df1*	*df2*	*P*
VF	Corrected model	7.538	4	504	≤0.001^*^
	Group size	0.014	1	504	0.905
	Reproductive status	13.589	2	504	≤0.001^*^
	Group size * reproductive status	1.119	1	504	0.291
MVD	Corrected model	4.912	4	504	0.001^*^
	Group size	4.937	1	504	0.027^*^
	Reproductive status	8.853	2	504	≤0.001^*^
	Group size * reproductive status	4.092	1	504	0.044^*^
PVF	Corrected model	9.911	4	504	≤0.001^*^
	Group size	1.933	1	504	0.165
	Reproductive status	16.5	2	504	≤0.001^*^
	Group size * reproductive status	1.836	1	504	0.176
PVI	Corrected model	3.249	4	590	0.012^*^
	Group size	0.659	1	590	0.417
	Reproductive status	5.891	2	590	0.003^*^
	Group size * reproductive status	1.643	1	590	0.200

^*^Significant difference at *P* < 0.05.

Vigilance during the lambing season was significantly greater than in the rutting season and non-reproductive season, but vigilance in the rutting season and non-reproductive season was not different (*post hoc* LSD test; Table 3).

**Table 3:** Pairwise comparisons of reproductive status on FGMs and vigilance of Przewalski’s gazelle

Pairwise contrasts		Estimate	*P*
FGM	Rutting–non-reproductive	−0.060	0.109
	Rutting–lambing	−0.270	≤0.001^*^
	Lambing–non-reproductive	0.210	≤0.001^*^
VF	Rutting–non-reproductive	−0.173	0.274
	Rutting–lambing	−0.835	≤0.001^*^
	Lambing–non-reproductive	0.662	≤0.001^*^
MVD	Rutting–non-reproductive	−8.003	0.085
	Rutting–lambing	−14.184	0.001^*^
	Lambing–non-reproductive	6.18	0.063
PVT	Rutting–non-reproductive	−0.046	0.007^*^
	Rutting–lambing	−0.114	≤0.001^*^
	Lambing–non-reproductive	0.068	0.009^*^
PVI	Rutting–non-reproductive	−0.026	0.205
	Rutting–lambing	−0.087	0.001^*^
	Lambing–non-reproductive	0.061	0.003^*^

^*^Significant level of *post hoc* pairwise comparisons was 0.05/3 = 0.0167.

FGM concentrations increased significantly with all the vigilance parameters: with vigilance frequency (*r* = 0.421, *P* = 0.032), with the number of vigilant individuals in the group (*r* = 0.405, *P* = 0.040), with MVD (*r* = 0.445, *P* = 0.023) and with the proportion of time spent vigilant (*r* = 0.441, *P* = 0.024).

## Discussion

Our study revealed that FGM concentration of individual female gazelles increased 4-fold after capture and transportation. Physiological response of elevated FGMs of the female gazelles under stressful conditions was successfully detected by our measure methods in this study.

FGM concentration and most vigilance behaviours did not differ between individuals in the large and small group of female Przewalski’s gazelles, with one exception—MVD. MVD was longer for individuals in the larger group. The ‘group-size effect’ on vigilance has been evidenced in many species of birds and mammals (Beauchamp, 2008). Previous studies have shown that vigilance rates in Przewalski’s gazelles differ with sex, reproductive status, group size, social rank and position within a group (Li *et al.*, 2009, 2012; Shi *et al.*, 2010b), and vigilance among females, but not males, follows the ‘group-size effect’ (Li *et al.*, 2009, 2012; Shi *et al.*, 2010b). This difference between sexes may reflect a corresponding difference in the function of vigilance behaviour between male and female gazelles (Li *et al.*, 2009; Shi *et al.*, 2010b). For females, vigilance is mostly directed towards predators, and is thus consistent with the ‘group-size effect’. By contrast, males’ vigilance is directed towards predators, as well as rival males and potential mates, and consequently, male vigilance does not follow the group-size effect (Shi *et al.*, 2010b). Such difference between the sexes has also been found in other ungulates, as well as in the occurrence of sex/age-specific group-size effect (Fattorini and Ferretti, 2019; Pecorella *et al.*, 2019). In this study, only female gazelles were sampled to exclude the confounding effects of sex.

The lack of predation pressure in the enclosure may explain the phenomenon that group size did not affect FGMs and vigilance of female gazelles in this study. Some species rapidly lose antipredator behaviours within generations (Jolly *et al.*, 2018), or adjust these behaviours to other functions such as intra-specific interaction or human avoidance (Zheng *et al.*, 2013). Our study population has been kept in an enclosure for one decade, and it is unlikely that their antipredator responses have been lost, but no group-size effect implies that such a possibility should be considered in *ex situ* conservation programs.

In our study, both the FGM concentrations and the vigilance level of female gazelles were greatest in the lambing season. Mothers with offspring are very vulnerable to predation because offspring are less able to detect and escape from predators (red deer *Cervus elaphus*: Childress and Lung, 2003; Przewalski’s gazelle: Li *et al.*, 2009; Apennine chamois *R. pyrenaica ornata*: Fattorini *et al.*, 2018; fallow deer *Dama dama*: Pecorella *et al.*, 2019; roe deer *Capreolus capreolus*: Fattorini and Ferretti, 2019). Consequently, mother gazelles spend extra time vigilant to protect their lambs and themselves. The lowest FGM concentration and vigilance level occurred in the rutting season, likely because of harsh winter conditions during the rutting season when gazelles spend more time feeding, as has been noted in other northern hemisphere ungulates (Li *et al.*, 2012). GCs regulate basic processes like metabolism and behaviour to meet the energy demands of routine activities (Crossin *et al.*, 2016; Casagrande *et al.*, 2018). At baseline levels, GC concentrations increase towards the end of gestation and lactation across a range of mammals, because the investment in reproduction requires additional energy costs (Pavitt *et al.*, 2016; Fattorini *et al.*, 2018). The peak of FGMs of the female gazelles occurred during the lambing season, possibly due to their increased baseline GC concentrations to meet their energetic demands for breeding.

FGM concentration increased as vigilance increased in this study. Because stressors can be highly variable, it is critical for animals to adjust their adrenal activities and behavioural strategies according to environmental conditions for survival, by enhancing detection of risk and predator avoidance while minimizing the costs of antipredation responses (Voellmy *et al.*, 2014; Zanette *et al.*, 2014). It is not possible to verify whether an increase in FGM concentration results in an increase in vigilance level, or vice versa, but FGMs and vigilance level are associated with the entire antipredator responses of female Przewalski’s gazelles. Vigilance of female gazelles was greatest in the lambing season when FGM concentration also increased, possibly due to increased baseline GC levels needed to meet the energy demands of lactation (Pavitt *et al.*, 2016; Fattorini *et al.*, 2018). This increase in FGM concentration could give rise to more vigilance and help females and their offspring get through periods when they are most vulnerable. Nonetheless, FGMs can be used as indicator of overall stress when direct predation is not easily observed, as in this study.

In conclusion, FGM concentrations and vigilance levels of individual free-ranging female Przewalski’s gazelles changed with their reproductive status, but not with group size. FGMs and vigilance level were correlated, suggesting that FGMs are a useful indicator of stress in female gazelles. From an animal welfare point of view, management should be adjusted according to the reproductive status of the gazelles, and in captive populations, stress factors should be minimized for females in the lambing season. These management measures could include lowering population density and providing more space or preparing shelters for mothers and lambs. To ensure that evolutionary selection pressures are maintained in the management of *ex situ* protection, it is important to consider predation pressure and vigilance behaviours to ensure the survival of endangered species when released back into the wild.

## Funding

This work was funded by the National Natural Science Foundation of China (project no.: 31572281) and the Interdisciplinary Research Funds of Beijing Normal University.

## Declarations of interest

none.
